# Privacy and Security in Multi-User Health Kiosks

**DOI:** 10.5195/ijt.2017.6217

**Published:** 2017-06-29

**Authors:** HAROLD TAKYI, VALERIE WATZLAF, JUDITH TABOLT MATTHEWS, LEMING ZHOU, DILHARI DEALMEIDA

**Affiliations:** 1 DEPARTMENT OF HEALTH INFORMATION MANAGEMENT, SCHOOL OF HEALTH AND REHABILITATION SCIENCES, UNIVERSITY OF PITTSBURGH, PITTSBURGH, PA, USA; 2 SCHOOL OF NURSING & UNIVERSITY CENTER FOR SOCIAL AND URBAN RESEARCH, UNIVERSITY OF PITTSBURGH, PITTSBURGH, PA USA

**Keywords:** Confidentiality, Multi-user health kiosk, Privacy, Risk assessment, Security

## Abstract

Enforcement of the Health Insurance Portability and Accountability Act (HIPAA) and the Health Information Technology for Economic and Clinical Health Act (HITECH) has gotten stricter and penalties have become more severe in response to a significant increase in computer-related information breaches in recent years. With health information said to be worth twice as much as other forms of information on the underground market, making preservation of privacy and security an integral part of health technology development, rather than an afterthought, not only mitigates risks but also helps to ensure HIPAA and HITECH compliance. This paper provides a guide, based on the Office for Civil Rights (OCR) audit protocol, for creating and maintaining an audit checklist for multi-user health kiosks. Implementation of selected audit elements for a multi-user health kiosk designed for use by community-residing older adults illustrates how the guide can be applied.

The shift toward adoption of electronic health records (EHRs) and various computer systems in healthcare has been motivated in part by the need to provide consumers and clinicians with timely access to protected health information (PHI) and decision support systems (Ballmann, 2015; Kokkonen et al., 2013; Kowitlawakul, Chan, Pulcini, & Wang, 2015; Rindfleisch, 1997). These technologies store and transmit large amounts of electronic protected health information (ePHI), necessitating vigilance in implementing protocols to optimize the privacy and security (P&S) of users’ data. Such action is especially important for blocking attempts to exploit the vulnerabilities of these systems and preventing unauthorized access to ePHI (Adhikari, Richards, & Scott, 2014; Gunter & Terry, 2005; O’Brien & Yasnoff, 1999).

Growing concerns over the P&S of healthcare information have brought about expansion of healthcare regulations such as HIPAA and HITECH to safeguard patient data/information. These concerns have also resulted in the overhaul of the P&S requirements necessary to achieve compliance, as well as tremendous increases in fines for noncompliance (Kwon & Johnson, 2013). Noncompliance with HIPAA can lead to severe consequences for covered entities (CEs). The most severe consequence is a fine of up to $250,000 and up to 10 years of imprisonment if the intent is to sell, transfer, or use individually identifiable health information for commercial advantage, personal gain, or malicious purposes (Annas, 2003; Choi, Capitan, Krause, & Streeper, 2006). This maximum fine has been increased to $1.5 million with the HITECH rule. Internal audit checklists can help to mitigate the security vulnerabilities of healthcare applications and technologies. By serving as the blueprint for broader and more detailed P&S policies, these checklists can be implemented in existing systems. Likewise, they can be incorporated into the development life cycle of new self-service systems and technologies, particularly those situated in the community outside of institutional systems.

## SELF-SERVICE TECHNOLOGIES

Self-service enables consumers to perform activities related to the provision of a particular service without the intervention of a service provider (Ding, Verma, & Iqbal, 2007; Yang, Lee, Park, & Lee, 2014). Self-service technologies (SSTs) contribute an estimated $130 billion to the U.S. economy and have been used successfully for years. This huge fiscal impact, coupled with advances in computer hardware, software, and Internet technology, means that SSTs are being deployed in more and more sectors of the service delivery system (Castro et al., 2010). Examples include automated teller machines (ATMs), flight check-in kiosks, pay-at-the-pump gas stations, self-pay parking meters and pay stations, CD rental kiosks, self-checkout kiosks at supermarkets, Internet and cell phone apps, and online classes or e-learning.

Many people use SSTs without even knowing it, as when they pay bills online or fill their gas tanks. The main factors driving adoption of SSTs across all major industries are efficiency and cost savings. While providing organizations with a competitive advantage (Hsieh, 2015) and enabling employees to perform other functions (Burkhart, 2012; Castro, Atkinson, & Ezell, 2010), SSTs allow consumers to participate in service delivery and enjoy convenience and control.

## MULTI-USER HEALTH KIOSKS AND POTENTIAL THREATS

Self-service technologies deployed in the health sector include multi-user health kiosks such as those for self-monitoring of blood pressure that are frequently located in pharmacies and grocery stores (Curran & Meuter, 2005; Meuter, Ostrom, Roundtree, & Bitner, 2000). Hospitals often deploy multi-user health kiosks to automate patient management services for admission, discharge, appointment scheduling, and patient check-in. They also leverage these technologies for the processing of co-payments, patient consent forms, and prescription refill requests, and for verification of insurance eligibility, often in different languages (Meuter, Ostrom, Roundtree, & Bitner, 2000; Soares et al., 2016).

Multi-user health kiosks present several P&S issues that need to be addressed The same characteristics that make these devices attractive for use in the self-service environment also render them vulnerable to P&S breaches (Günay, Erbuğ, Hekkert, & Herrera, 2014; Smith, 2008; Uhley, 2006). Owing to their quasi-portable and unattended nature, multi-user health kiosks are typically deployed in public places. This makes them susceptible to invasion of privacy by bystanders as well as intrusion attacks by malicious individuals for whom unsupervised access provides cover for launching repeated attempts to breach kiosk systems.

Most kiosk patrons do not need explicit IT or network privileges such as user names and passwords to initiate interaction with the kiosk. They instead use some form of generic log-on information, which makes it challenging for system administrators to manage or track user activities and protect against security threats. Kiosk users can also become victims of identity theft and fraud if they are oblivious to “shoulder surfing” by others while logging in or entering information (Ciampa, 2008; Craig, 2008; Kizza, 2013b; Smith, 2008; Uhley, 2006).

Vandals can intentionally damage or compromise kiosk hardware by attaching their own devices to the network via accessible CD-ROM drives and USB ports, thereby instigating a man-in-the-middle attack. Because multi-user health kiosks are usually connected to larger, shared organizational networks (i.e., the same networks used for other information technology services), attackers can wreak considerable havoc on an organization’s network by compromising kiosks on that network. Attackers bent on bypassing kiosk operating system access controls can then access the underlying operating system and file system (Ciampa, 2008; Craig, 2008; Kizza, 2013b; Smith, 2008; Uhley, 2006).

Because multi-user health kiosks are used with little or no supervision, it is essential for them to be configured to prevent users from viewing others’ data, installing malicious programs, tampering with the kiosk software, or gaining access to the operating system and the file system. It is, however, very difficult to tie down systems without losing some of functionality. A balanced approach to mitigating P&S risks is the best way to go, and it should include these steps:

1. Deploy multi-user health kiosks in well-lit areas, to protect both the user and the equipment from violent or malicious people.
2. Install privacy screens on kiosks, to make it difficult for anyone else to see what appears on the screen when someone is logged on.
3. Prevent unrestricted access to the underlying kiosk hardware by eliminating external access to cabling or internal components such as hard drives and USB and serial ports that would allow installation of malicious software or devices.
4. Enclose internal components including hard drives in secure housings to prevent theft of hardware.
5. Avoid peripheral devices such as keyboards that could enable hackers to install devices like keyboard recorders to capture users’ keystrokes and thus gain access to personal and confidential information.
6. Equip each kiosk with a touch screen instead of a regular keyboard and mouse (if possible). If a physical keyboard is unavoidable, opt for a special keyboard without function keys.
7. Deploy kiosks on their own dedicated networks, and utilize sub-netting, firewalls, and other intrusion prevention systems in order to segment the kiosk networks from other networks used by the organization.
8. Use special-purpose operating systems specifically designed for kiosks to prevent users from performing unauthorized functions.
9. Configure the operating system access control mechanism to make it difficult to bypass, by using “reference monitoring,” or a set of well-defined design requirements, to enforce the access control mechanism (Craig, 2008; Jaege, 2013).

## TRICKS BY ATTACKERS

Kiosks in general may be exposed to a host of network attacks. The following are tricks that attackers may employ to get around kiosks’ access control mechanisms:

- Most health kiosks use Microsoft applications that have built-in Visual Basic (VB) editors. Attackers can activate and use these editors to write small scripts to open loopholes by which to gain unlimited access to the system. For example, an attacker can use the ALT+F1 key combination in a blank Word document to open up the VB editor. Similar tricks can be employed in the VI text editor in Linux (Ballmann, 2015; Craig, 2008).
- Browsers offer another way an attacker can gain access to the file system. Most kiosks have various functionalities of browsers disabled. Typically, the address bar is disabled. However, holding down the shift key and clicking on a hyperlink will open up the link in a new browser window, usually with the address bar enabled (Craig, 2008).
- The calculator provides access to another method an attacker can use to infiltrate a kiosk system. Most health kiosks use Microsoft Windows operating systems that contain calculators. Clicking F1 while the calculator application is open will usually activate the Help function. There is a tap in the Help function labeled ‘Jump URL.” Clicking on this will open the web browser and provide access to other areas of the file system (Craig, 2008).

Additional security concerns pertain to kiosks designed and deployed in the context of healthcare. Examples include:

- Masquerading/unauthorized access: By gaining unlawful access to another user’s credentials through illegal means such as hacking or shoulder surfing, imposters can gain access to that user’s health data or escalate their privileges on a network (Ballmann, 2015; Craig, 2008).
- Unauthorized use of resources: Unscrupulous users can utilize various illegal means including privilege escalation, backdoors, rootkit, default accounts, and unprotected access points to gain access to resources on a network or network computers, allowing them access to another user’s PHI (Ballmann, 2015; Craig, 2008).
- Unauthorized disclosure and flow of information: Once an attacker has access to the kiosk system, he or she can install network taps or malicious code/applications to gain access to a host of personal information, including information retained on kiosks or saved on servers and other network devices. After obtaining this initial information, the attacker can engage in further clandestine activities such as man-in-the-middle attacks and denial-of-service attacks (Ballmann, 2015; Smith, 2008; Smith, 2012; Uhley, 2006).
- User errors/forgetfulness: The least talked-about P&S vulnerability of healthcare kiosks is failure by a user to log out completely or to exit the system after using it. This is an easy setup for another person to latch onto the non-terminated session to gain access to the user’s information or even compromise the entire system (Fei Yu, 2011; Kizza, 2013a).

For multi-user health kiosks to be HIPAA/HITECH-compliant and meet the requirements of other state and federal regulations, procedures must be in place to minimize P&S threats. In the absence of clear-cut compliance measures, kiosk architecture should be designed from the bottom up with HIPAA/HITECH and other regulations in mind. That means that the system should be able to protect or ensure security, privacy, confidentiality, integrity, availability, and non-repudiation of information. Careful attention must also be paid to aspects of HIPAA/HITECH that deal with CEs and business associates (BAs). Audit checklists based on the OCR audit protocol should be incorporated into the development and deployment process of health kiosks.

## DEVELOPING A PRIVACY AND SECURITY CHECKLIST FOR A MULTI-USER HEALTH KIOSK

The Health Kiosk Project at the University of Pittsburgh provides an example of how such an audit checklist has been developed. Funded by the Agency for Health Care Research and Quality (5R01HS022889 PI: Matthews), the project involves several health kiosks that have been designed for use by older adults in community-based congregate settings. The settings include senior centers, subsidized senior housing, and continuing care retirement communities.

Each kiosk consists of a wheeled desk and desk chair, touch screen monitor, RFID reader, printer, and selected medical devices that either require manual entry of measurements (blood pressure monitoring device) or are integrated (hand dynamometer and seated scale) with the on-board computer. The hard drive is encrypted, as are data transferred from the kiosk via MiFi hotspot to secure university servers. A cell phone in the kiosk drawer facilitates users’ requests for assistance, and a messaging feature on the touch screen enables textual communication with the project team.

At the kiosk, users self-administer health-related surveys, learn behavioral strategies for improving aspects of their health, and receive graphical feedback depicting their progress toward personal goals related to sleep, bladder control, mobility, and mood, among other topics. Wireless headphones convey voiceover for all content displayed on the touch screen. Relevant educational materials may be printed to take home.

The following steps were implemented to develop an audit checklist for addressing potential P&S vulnerabilities of the kiosks in the Health Kiosk Project:

1. Investigate and Research Possible Security Vulnerabilities: This step entailed garnering expert opinions from published work, textbooks, and interviews with people involved in the design and development of the system, and from “walking through the systems” (Bishop, 2003; Craig, 2008; Garg & Camp, 2015). Specifically, we drew from the literature, interviews with the project team, and direct interaction with the kiosk. We also used the penetration testing techniques (PENTESTING) specified by Craig (2008) to aid in identifying possible vulnerabilities of our multi-user health kiosk design.
2. Perform a Risk Assessment: Eight steps were involved in assessing the extent to which P&S could be breached (Appari & Johnson, 2010; Oyelami & Ithnin, 2015; Stoneburner, Goguen, & Feringa, 2002):
  - Characterize the system: This step helped to define the scope of the risk assessment by identifying items that needed to be protected. We recognized that a solid understanding of the system’s architecture as a whole was needed to successfully complete this step (Garg & Camp, 2015; Oyelami & Ithnin, 2015). Hence, system information was collected and classified as: hardware, software, system interfaces (external and internal connectivity), data and information, individuals who support as well as use the system, main functions of the system (functions performed by the system), criticality of the various components of the system to the organization (e.g., how critical the particular component is to system functionality), and sensitivity of system components. After carefully looking through and analyzing various aspects of the health kiosk system, working with the project team, and using information about P&S for multi-user health kiosks discussed earlier in this paper, we identified areas of the system that needed to be protected. These areas formed the core part of the header for the major sections of our audit checklist.
  - Identify threats: Possible threats to the system that could lead to vulnerabilities were characterized as high, medium, or low. Informed by expert opinion, the developer’s past experience, and industry trends and standards, we focused on identifying anticipated threats rather than every possible threat, as the latter could have been overwhelming and unrealistic to accomplish (Gribaudo, Iacono, & Marrone, 2015; Oyelami & Ithnin, 2015). We used this process to decide which aspects of P&S were worth protecting. Again, information pertaining to possible threats to kiosks in general, physical interaction with the kiosk during development, and discussions with the project team were instrumental in identifying the sources of threat to our multi-user health kiosk.
  - Identify vulnerabilities: Action must be taken to identify the vulnerabilities that can result from threats because vulnerabilities suggest possible weaknesses in the system that can be exploited by adversaries bent on breaching the system. Some of the ways to identify vulnerabilities are system security testing and evaluation, penetration testing, and vulnerability scanning using any type of automated vulnerability testing tool (Rebollo, Mellado, Fernández-Medina, & Mouratidis, 2015; Rinehart-Thompson, 2013). We undertook this step in discussion with the main developer of the kiosk to identify whether vulnerabilities existed pertaining to password protection, privilege escalation, applications and user authentication, and encryption, to mention but a few.
  - Control and analysis: This step entails reviewing and analyzing controls that have been implemented or are planned to be implemented, to reduce the probability of a threat or adversary exploiting the system. As part of this step, impact analysis should be performed to determine the impact (i.e., loss of integrity, loss of availability, and loss of confidentiality) to the system in case a vulnerability is exploited. The controls can be technical or non-technical. An example of a technical control would be implementing an encryption strategy to protect data. Non-technical controls could include personnel training regarding proper methods for reducing the probability of a vulnerability occurring. Means of control should be preventive, deterrent, detective, reactive, and capable of recovery (Rebollo et al., 2015; Rinehart-Thompson, 2013).
    The Health Kiosk Project team considered the impact that the identified vulnerabilities could have on the functionalities of the kiosk. The team then acted to minimize or eliminate those vulnerabilities that posed the greatest risk.
  - Determine likelihood of occurrence. This step involves estimating the likelihood (high, medium, or low) that a particular vulnerability will occur (Rinehart-Thompson, 2013). The Health Kiosk Project team examined the design and types of activities performed on the kiosk to further decide which vulnerabilities were more likely to occur. This resulted in further streamlining of the kiosk features and functionalities that we wanted to protect to include in our audit protocol.
  - Determine risk: Assessing the level of risk to the IT system allows for expression of the level of threat and vulnerability for the pairs that have been identified, the magnitude of the impact in the event that a vulnerability is successfully exploited by a given threat, and determination as to whether adequate P&S procedures have been put in place to reduce the risk (Nazareth & Choi, 2015; Rinehart-Thompson, 2013). For the Health Kiosk Project, we had a series of meetings to discuss how the different vulnerabilities could impact the functionality of the kiosk, including what would happen if there were no backups and data were corrupted or lost in the backend database, or whether there was a redundant power supply in case of power outages.
  - Recommend controls: To reduce or eliminate perceived risk, recommendations need to be enacted that are appropriate for an organization’s operations, requirements, legislated mandates, and standards. Factors that should be considered during this process include, but are not limited to, effectiveness of the recommended options such as system compatibility, legislation and regulation, organizational policy, operational impact, and safety and reliability (Rinehart-Thompson, 2013). The Health Kiosk Project team used information gathered in the earlier steps as well as requirements for HIPAA and HITECH compliance to decide the aspects of the OCR audit checklist to incorporate into our final audit checklist.
  - Document the result: Threat sources and potential vulnerabilities that are identified should be documented in a report or briefing (Rinehart-Thompson, 2013). For our work, we matched the potential vulnerabilities to the OCR Audit protocol. We then adopted aspects of the OCR audit protocol that match our vulnerabilities to develop an audit checklist for the multi-user health kiosk (Appendix A) which can be used by any developer, researcher, or other user of the health kiosk to make sure that the system meets the P&S provisions.
3. Specify the Checklist: The audit checklist was then finalized for our kiosk by adapting parts of the OCR audit checklist, a checklist developed by Watzlaf et al., and a Security Self-Assessment Guide for Information Technology Systems that was developed by the National Institute of Standards and Technology (Christiansen, 2013; Swanson, 2001; Watzlaf, Moeini, & Firouzan, 2010; Watzlaf, Moeini, Matusow, & Firouzan, 2011).

## CONCLUSION

Recent increases in privacy and security breaches as well as increased oversight and fines for HIPAA and HITECH violations (Solove, 2013) underscore the need for a rigorous approach to ensure that adequate P&S protections are in place in self-service technologies that involve personal health information. Securing information technology systems such as those involved in multi-user health kiosks is usually an afterthought in system development. The process for checklist development discussed in this article can help to make P&S protections part of the system development life cycle. The checklist can also be used in the development of P&S policies. Recognizing that there cannot be HIPAA and HITECH compliance without P&S policies (Maji et al., 2008; Peterson & Watzlaf, 2015), we endeavor to address that challenge in relation to multi-user health kiosks. We maintain that having a comprehensive audit checklist for health technologies can help with HIPAA and HITECH compliance.

## APPENDIX A MULTI-USER HEALTH KIOSK AUDIT CHECKLIST

The protocol below provides a guideline that can be used to assess whether a multi-user health kiosk is meeting privacy and security (P&S) regulations such as HIPAA and HITECH. It has been adapted from the OCR audit protocol and the checklists developed by Watzlaf et al. (2010; 2011; 2012), Peterson and Watzlaf (2014), Swanson (2001), and Watzlaf et al. (2010).

**Table:**

HIPAA/HITECH Compliance Checklist for Multi-User Health Kiosk			
**PRIVACY**	Yes	NO	N/A
1. Personal Information (§164.506, §164.514 (Swanson, 2001; Watzlaf et al., 2010)			
Is there a privacy policy?			
Does the kiosk have a privacy screen?			
Will user information be shared with third-party organizations?			
○ If yes, is there a Business Associate agreement (BAA) with this organization?			
2. Retention of Personal Information			
Is user information and e-PHI stored?			
Is there a policy outlining the retention period of e-PHI?			
Can users request copies of their information?			
○ If yes, is there a well-defined procedure for requesting copies of PHI and other information?			
**CONFIDENTIALITY** §164.522 (Swanson, 2001)			
3. Request of Information			
Is there a policy for disclosure of e-PHI or identifiable information?			
**SECURITY** §164.308 (Swanson, 2001; Watzlaf et al., 2010; Watzlaf et al., 2011)			
4. Security Management Process			
Is there a well-written procedure or protocol for performing a thorough risk assessment?			
How many times a year is a risk assessment performed? ○ 0 times per year ○ Once a year? ○ Twice a year? ○ Three times a year? ○ More than three times a year?			
Is there a formal or informal policy or procedure to review information system activities like audit logs, access reports incident tracking etc.?			
Are current security measures sufficient to reduce risk and vulnerabilities to a reasonable level?			
5. Assigned Security Responsibility			
Do you have a security officer in charge of developing, implementing, monitoring and communicating HIPAA/HITECH security policies and procedures?			
6. Workforce Security			
Do you have documentation for authorization and supervision of all entities working with or helping to manage and maintain the kiosk?			
Do you have clear job descriptions for all entities working with the kiosk?			
Is there documentation listing the level of access to the system, including e-PHI for each employee?			
Is there a clear procedure to terminate access to resources once a person is removed from the project or terminated?			
7. Information Access Management			
Is there a clear written procedure to grant access to e-PHI?			
Do policies and standards exist to authorize and document access, review and modify a user’s right to computer systems, software, databases and other network resources?			
Are users going to pay to use the kiosk system? ○ If so, will a clearinghouse or third party be used to process payment? ▪ If so, are there policies and procedures for access to information, by clearinghouse workers, consistent with HIPAA and HITECH security rules?			
Are formal or informal policies and procedures in place for security measures relating to access control?			
Is there any HIPAA and HITECH security awareness and training program in place?			
Are there procedures and measures in place for protection from malicious software and exploitation of vulnerabilities?			
Have employees been trained as to the importance of protecting against malicious software and how to guard against it?			
Are there policies and procedures for log–on monitoring and password management?			
Do security training materials target current IT security topics relevant to kiosk security?			
How often are security procedures, policies and protocols updated? ○ 0 times per year? ○ Once a year? ○ Twice a year? ○ Three times a year? ○ More than three times a year?			
Are there any policies and procedures in place to identify, respond to, report and mitigate security incidents?			
8. Contingency Plan			
Is there a contingency plan in place to identify critical applications, data and other operations of the kiosk system?			
Is there a disaster recovery and backup plan in place to restore lost data?			
Is any redundancy built into the kiosk deployment?			
Is there any well-defined policy for operating in emergency mode that allows continuation of critical business processes?			
Are there any policies for testing emergency contingency plans or backup procedures?			
9. Evaluation			
Are there policies in place for evaluating the security procedures as they apply to HIPAA/HITECH security rules?			
10. Business Associate (BA) Contracts			
Is there a policy for contracts with Business Associates and other third-party vendors?			
11. Physical Security			
Are there policies in place to analyze physical security vulnerabilities of the kiosk system?			
Are there policies in place to guard against physical security vulnerabilities and to protect kiosk hardware and components that hold e-PHI?			
Are there procedures and policies in place to control access to kiosk hardware, systems and other components by staff, visitors etc. that could compromise the kiosk system as a whole?			
Are there maintenance records for repairs and modification of physical components especially relating to security?			
12. Computer Component Use			
Is there other computer hardware, like workstations and servers that manage the kiosk system?			
○ If yes, are there policies and documentation outlining specific workstations and servers and their functions and location?			
○ Is there documentation and procedures to identify specific functions of each workstation and server?			
13. Workstation and Server Security			
Is there any policy or procedure to prevent unauthorized access to an unattended workstation or to limit the ability of un-authorized persons to access other users’ information (analyze physical surroundings for physical attributes)?			
How are workstations and servers physically restricted to limit or restrict access to only authorized people?			
14. Device and Media Controls			
Is there any policy for monitoring and tracking the location and movement of kiosk hardware (especially containing e-PHI)?			
15. Access Control			
Is there an access control policy?			
Is there an encryption procedure in place to protect e-PHI?			
○ If yes, are there any well documented policies governing and outlining the encryption strategy?			
Are there any policies to make sure all users are assigned unique access credentials, like IDs and passwords, to log on to the kiosk system?			
Are all users assigned usernames and passwords?			
Is there documentation of each user’s exact privileges in the kiosk system (useful to prevent privilege escalation)?			
Are there clearly defined policies to track changes and modifications made within the kiosk system, including which users made the changes?			
Are there any policies in place to make sure user access is reviewed on a periodic basis and how often that is done?			
Is the system configured to auto-logoff after a predetermined time?			
○ Is there any documentation and defined policy for this?			
Are there procedures for terminating access when it is no longer needed?			
16. Audit Control			
Has any audit control been implemented?			
Are there any audit control policies in place?			
How often are the audit control tools and mechanisms reviewed to determine if upgrades are needed? ○ 0 times per year? ○ Once a year? ○ Twice a year? ○ Three times a year? More than three times a year?			
Integrity			
Who has access to information or e-PHI stored in the kiosk systems?			
Is there a well-defined policy or procedure to identify these individuals?			
Person or Entity Authentication			
What kind of authentication procedure or mechanism is in place within the kiosk system?			
Are there any policies to govern this and also evaluate the authentication mechanisms in place to assess the strengths and weaknesses of the mechanism?			
If so does the policy also look at the cost benefit ratio of the various types of authentication mechanisms?			
Is there a policy to test and upgrade the authentication mechanism tested on a periodic basis?			
Transmission Security			
Is there any formal data transmission policy for the kiosk system?			
Is there any risk assessment policy to determine the security level of the data transmission procedure in the kiosk system?			
Is there a formal policy for breach notification?			
Is there a template or letter or other defined means of breach notification?			
Does the notification policy include procedure for notification of media outlets?			
Does the policy also spell out notification procedures for Business Associates, if any?			

## References

[b1-ijt-09-03] Adhikari R, Richards D, Scott K (2014). Security and privacy issues related to the use of mobile health apps. http://www.colleaga.org/sites/default/files/attachments/acis20140_submission_12.pdf.

[b2-ijt-09-03] Annas GJ (2003). HIPAA regulations—a new era of medical-record privacy?. New England Journal of Medicine.

[b3-ijt-09-03] Appari A, Johnson ME (2010). Information security and privacy in healthcare: Current state of research. International Journal of Internet and Enterprise Management.

[b4-ijt-09-03] Ballmann B (2015). Understanding network hacks.

[b5-ijt-09-03] Bishop M (2003). What is computer security?. Security & Privacy, IEEE.

[b6-ijt-09-03] Choi YB, Capitan KE, Krause JS, Streeper MM (2006). Challenges associated with privacy in health care industry: Implementation of HIPAA and the security rules. Journal of Medical Systems.

[b7-ijt-09-03] Christiansen JR (2013). HIPAA/HITECH Compliance: Using the OCR audit protocols.

[b8-ijt-09-03] Ciampa M (2008). Security+ Guide to Network Security Fundamentals, 1 yr.

[b9-ijt-09-03] Ballmann Big P (2008). Hacking internet kiosks.

[b10-ijt-09-03] Ding X, Verma R, Iqbal Z (2007). Self-service technology and online financial service choice. International Journal of Service Industry Management.

[b11-ijt-09-03] Fei Yu RJ (2011). Mobile device security.

[b12-ijt-09-03] Garg V, Camp L (2015). Risk characteristics, mental models, and perception of security risks.

[b13-ijt-09-03] Gribaudo M, Iacono M, Marrone S (2015). Exploiting Bayesian networks for the analysis of combined attack trees. Electronic Notes in Theoretical Computer Science.

[b14-ijt-09-03] Günay A, Erbuğ Ç, Hekkert P, Herrera NR (2014). Changing paradigms in our interactions with self-service kiosks. Human-Computer Interfaces and Interactivity: Emergent Research and Applications: Emergent Research and Applications.

[b15-ijt-09-03] Gunter TD, Terry NP (2005). The emergence of national electronic health record architectures in the United States and Australia: Models, costs, and questions. Journal of Medical Internet Research.

[b16-ijt-09-03] Hsieh C-t (2015). Implementing self-service technology to gain competitive advantages. Communications of the IIMA.

[b17-ijt-09-03] Kizza JM (2013a). Computer network vulnerabilities. Guide to computer network security.

[b18-ijt-09-03] Kizza JM (2013b). Security threats to computer networks. Guide to computer network security.

[b19-ijt-09-03] Kokkonen EWJ, Davis SA, Lin H-C, Dabade TS, Feldman SR, Fleischer AB (2013). Use of electronic medical records differs by specialty and office settings. Journal of the American Medical Informatics Association.

[b20-ijt-09-03] Kowitlawakul Y, Chan SWC, Pulcini J, Wang W (2015). Factors influencing nursing students’ acceptance of electronic health records for nursing education (EHRNE) software program. Nurse Education Today.

[b21-ijt-09-03] Kwon J, Johnson ME (2013). Security practices and regulatory compliance in the healthcare industry. Journal of the American Medical Informatics Association.

[b22-ijt-09-03] Meuter ML, Ostrom AL, Roundtree RI, Bitner MJ (2000). Self-service technologies: Understanding customer satisfaction with technology-based service encounters. Journal of Marketing.

[b23-ijt-09-03] Nazareth DL, Choi J (2015). A system dynamics model for information security management. Information & Management.

[b24-ijt-09-03] O’Brien DG, Yasnoff WA (1999). Privacy, confidentiality, and security in information systems of state health agencies. American Journal of Preventive Medicine.

[b25-ijt-09-03] Oyelami JO, Ithnin NB (2015). Establishing a sustainable information security management policies in organization: A guide to information security management practice (ISMP). International Journal of Computer and Information Technology.

[b26-ijt-09-03] Rebollo O, Mellado D, Fernández-Medina E, Mouratidis H (2015). Empirical evaluation of a cloud computing information security governance framework. Information and Software Technology.

[b27-ijt-09-03] Rindfleisch TC (1997). Privacy, information technology, and health care. Communications of the ACM.

[b28-ijt-09-03] Rinehart-Thompson LA (2013). Introduction to health information privacy and security.

[b29-ijt-09-03] Smith B (2008). Hacking the kiosk.

[b30-ijt-09-03] Smith G (2012). White house hacked in cyber attack that used spear-phishing to crack unclassified network.

[b31-ijt-09-03] Soares E, Oliveira C, Maia J, Almeida R, Coimbra M, Brandão P, Prior R (2016). Modular health kiosk for health self-assessment.

[b32-ijt-09-03] Solove DJ (2013). HIPAA turns 10: Analyzing the past, present and future impact. Journal of AHIMA.

[b33-ijt-09-03] Stoneburner G, Goguen A, Feringa A (2002). Risk management guide for information technology systems. NIST Special Publication.

[b34-ijt-09-03] Swanson M (2001). Security self-assessment guide for information technology systems. NIST Special Publication.

[b35-ijt-09-03] Uhley P (2006). Kiosk security.

[b36-ijt-09-03] Watzlaf VJ, Moeini S, Firouzan P (2010). VoIP for telerehabilitation: A risk analysis for privacy, security, and HIPAA compliance. International Journal of Telerehabilitation.

[b37-ijt-09-03] Watzlaf VJ, Moeini S, Matusow L, Firouzan P (2011). VOIP for telerehabilitation: A risk analysis for privacy, security and HIPAA compliance: Part II. International Journal of Telerehabilitation.

[b38-ijt-09-03] Yang H-D, Lee J, Park C, Lee K (2014). The Adoption of Mobile Self-Service Technologies: Effects of Availability in Alternative Media and Trust on the Relative Importance of Perceived Usefulness and Ease of Use. International Journal of Smart Home.

